# Transactions of the Medical Society of the State of New-York. Vol. VI. Part III. 1846

**Published:** 1846-06-01

**Authors:** 


### ECLECTIC DEPARTMENT,

### AND SPIRIT OF THE MEDICAL PERIODICAL PRESS.

*Transactions of the Medical Society of the State of New-York. Vol. VI. Part III.* 1846.

We have received the Transactions of the State Medical Society, for the current year, from some unknown friend, who will please accept our acknowledgements. They come with an improved external aspect, and if the typography be not symbolical of advancement in other respects, it is, at least, certain that the published papers compare very favorably with those of the last and preceding numbers. We proceed to give some account, in the first place, of these, and afterward of the proceedings of the Society.

PHLEBITIS: —*An Address read at the Annual Meeting of the Medical Society of the County of Albany; by Thomas Hun, M. D., President of the Society.*

This is a valuable practical memoir of a highly interesting and important subject. It relates to a disease of not very unfrequent occurrence, which was not well understood until lately, and chiefly through the investigations of Dance, Velpeau and Cruveilhier. The latter has devoted attention particularly to it, and his great work on Morbid Anatomy contains some beautiful illustrations of the appearances found after death.

Prof. Hun adopts Cruveilhier's division of the disease, into I, Adhesive Phlebitis; II, Suppurative Phlebitis.

*Adhesive Phlebitis.* The first effect is to cause the formation of a coagulum obstructing the circulation through the affected vessel. It is impossible, in the existing state of our knowledge, to explain the rationale of this effect. The probable explanation is, in so far as it goes, that the nervous energy in the part is diminished or perverted. The clot becomes fibrinous, and finally organised, so that the vessel is converted into a solid cord; or, on the other hand, it may, when limited and small in amount, be washed away, and the circulation through the vessel restored.

This variety of phlebitis generally occasions but little or no constitutional disturbance. The morbid effects are confined to disturbances due to the mechanical obstruction of the circulation. The latter will depend upon the size and extent of the venous trunks affected, the organ in which they are seated, the freedom of anastomosis, &c.

Prof. H. regards phlebitis as the proximate cause of *Phlegmasia alba Dolens.* The uterine veins, especially where the placenta was attached, become inflamed precisely as occasionally after wounds or surgical operations. The superficial veins in this affection frequently, as the swelling subsides, become largely developed, so as to establish a collateral circulation. This is a favorable result. The left limb is most frequently attacked. This is attributed to the pressure of the occiput of the child during labor upon the left side of the pelvis from its position in a natural presentation.

One of the most serious forms of adhesive phlebitis is where it attacks the sinus of the dura mater. It produces cerebral congestion, and death by interrupting the circulation of the brain.

*Suppurative Phlebitis.*—The name denotes the circumstance which distinguishes this variety, viz: the formation of pus. This is apt to occur under peculiar conditions of constitution, and certain external influences. The effluvia of large cities, and of crowded hospitals, are among the circumstances which favor it. Prof. H. speaks of the pus as secreted by the inflamed walls of the vessel in all cases. This may be so; but we think that it may be accounted for, in some instances, at least, by a conversion of the fibrinous coagula into this morbid product.

When suppuration takes place, it is a matter of great moment whether the pus be limited to a certain space, i. e. confined, as in ordinary abcess, by the adhesive inflamation at either extremity of the vein; or, on the other hand, whether it become commingled with the blood, and circulate in combination with this fluid. In the former case, the disease is comparatively trifling—it is not more serious than ordinary adhesive phlebitis. In the latter case, the effects are of a very grave character, and the result is generally fatal.

The presence of pus globules in the blood is not rendered certain, as yet, by microscopic observation. Several observers have felt confident of their having detected them, but there is room for the suspicion that they may have mistaken fibrinous concretions for pus.

A characteristic effect of prevalent infection of the blood is supposed to be the formation of abcesses, or the deposition of pus in various organs simultaneously. It is contended by some that these local deposits consist of the pus formed at the original source of the infection, which is carried along the circulation and deposited in various structures. This, however, is not probable. It is more rational to regard them as secondary morbid products.

The symptoms of purulent infection of the blood Prof. H. quotes from Berard, as follows:

"It is announced by a violent chill more or less prolonged. The feeling of cold is intense. To this succeeds a marked heat and reaction, followed by a sweat which is viscous and not critical. The chills are renewed within twenty-four hours, and afterwards several succeed each other at intervals. It is only in the first days that the chills are intense; they are afterward transient or even entirely wanting. The pulse is always very frequent, its other characters are variable; toward the end it becomes small and thready."

The patients are often delirious, and are unconscious of the danger of their situation. They suffer little.

"At an advanced period the skin takes a yellowish tinge as in jaundice, but the other signs of jaundice are wanting. The urine and other excretions are fetid; diarrhœa is often present. Purulent collections sometimes form in the cellular tissue; at other times pus collects in some of the articulations. Sometimes there are symptoms and signs indicating the formation of abcesses in the viscera, but in general they are difficult of detection. The abcesses of the lungs are too small to give rise to any decided signs by auscultation, or to produce much flatness by percussion. They are accompanied by a slight cough, and sometimes by a rusty expectoration. When the metastasic abscesses have caused an inflammation of the serous membrane covering the organ in which they exist, it is then possible to detect the presence of this inflammation. Such cases are common. As to the progress of the disease it is generally rapid. Death occurs in eight, ten or twelve days, but some resist the disease during several weeks. The rapidity of the accidents is in proportion to the quantity of pus which has passed into the veins. Sometimes, at the end of twenty-four or thirty-six hours, there is an apparent improvement, which is very apt to deceive."

Suppurative phlebitis followed by infection of the blood may arise from several remote causes. Among these are venesection, ligation of the veins, surgical operations, wounds or injuries, and parturition. When the cause does not act directly on the coats of the vein, as it does in venesection and ligation, it is an interesting question how does the pus get into the vessels to be commingled with the blood. The greater size of the pus globules when compared with the blood disks precludes the idea that they can pass through the coats of the vessels by transudation, or that they are capable of being absorbed in their organised state. It is thus explained by Douce and Cruveilhier: we quote the language of Prof. H. in giving an exposition of their views:

"The veins around a suppurating surface are necessarily involved in the inflammation. In ordinary cases, this inflammation is of the adhesive character, and hence no general disturbance is occasioned by it. But under miasmatic influences, and in certain states of the constitution, there is, as already stated, a tendency in all inflammations to run into suppuration. Hence, in these circumstances the inflamed veins around the suppurating surface secrete pus, which passes into the mass of the blood, as in the cases of inflammation of the larger veins. There is, therefore, no essential difference between the mode of production of the purulent infection of the blood from a wound or suppurating surface, and that which follows the ligature or other injury of a large venous trunk. Phlebitis necessarily exists around a suppurative surface, and accordingly as it is of the adhesive or suppurative character will it be harmless or fatal."

The following paragraph we quote as involving a question of great interest and importance:

"There is yet another mode in which this accident may be produced. As has been before remarked, parturition is always followed by phlebitis, which, in ordinary cases, passes off without notice. In other cases, it extends to the veins of the pelvis, and even of the extremeties, and causes phlegmasia alba dolens. *Again, in other cases, in consequence of the bad air of hospitals, or other causes this phlebitis passes to suppuration, purulent infection of the blood follows, and then we have the most fatal form of puerperal fever.*"

We have italicised the last sentence, in order to direct the reader's attention particularly to it. Prof. H. does not enlarge upon the subject, but disposes of it in the single sentence quoted. Are puerperal fever and suppurative phlebitis one and the same affection? This is a question which many will answer promptly in the negative, and which many more will hesitate to answer affirmatively from the absence of demonstrative facts sufficient to warrant the induction of the two diseases under a common pathological principle. There is in both instances a deficiency of direct positive facts with regard to the morbid state of the blood. No one has yet demonstrated in what mode the blood becomes diseased by the contamination of pus — in what consists the morbid alteration that ensues, and the connection which the perversion has with the ulterior consequences. Until these points are determined with respect to cases of acknowledged phlebitis, it is not, of course, to be expected that the identity of puerperel fever with it can be established by adducing a positive similarity in the essential morbid elements. But in so far as we are authorised to reason from analogy, and concurrent circumstances, in the existing state of our knowledge, it must be admitted that there are cogent arguments in favor of regarding both as in fact one disease. Take the description of phenomena characterising the presence and march of suppurative phlebitis as briefly sketched in the paper now referred to, and as more fully detailed by other writers, and it will be found to apply in almost every particular to the phenomena of epidemic puerperal fever. The one might, without much effort at accommodation, be substituted for the other. This strong analogy must strike every observer who has had an opportunity of comparing the two diseases. An opportunity once occurred to us to witness a fatal case of phlebitis following venesection, and the resemblance in general features pressed itself forcibly on our attention.

Admitting the correctness of the explanation submitted by Douce and Cruveilhier, of the manner in which the mass of the blood becomes infected from a suppurating surface, it is easy to conceive of its application to the placental portion of the uterus after parturition.

Another strong collateral circumstance is the fact that puerperal fever occurs, especially in its epidemic form, at periods when we have abundant evidence, apart from the fever itself, of the prevalence of a morbid condition of the system characterised by the absence of plasticity in local inflammations. The co-existence of erysipelas is evidence of it. And, again, the postmortem appearances in subjects dead of the disease in question, evince the same thing — i. e. a *diffusive* character of inflammation, and a want of plasticity will readily account for the absence of adhesive inflammation in the diseased veins and the consequent introduction of pus into the mass of the blood. But we cannot enter upon a discussion of this point at this time. Our only object is to direct the reflections of our readers in relation to it. The question, like all other similar questions, is to be definitively settled by facts, and facts only. But, unfortunately, these facts are yet to be developed. In the mean time, as the subject has practical relations, and we are called upon to act in conformity with what rational convictions we have, it behooves us to consider it with what light we may, and adopt the conclusions which, in the existing state of knowledge, seem nearest the truth. And we are free to express our conviction that puerperal fever involves as its chief pathological element a perversion of the blood; and that with our knowledge of what appertains to certain forms of phlebitis, we are safest in regarding the two diseases as very similar, and shaping our practical course accordingly.

Prof. H. concludes his paper by pointing out a morbid condition arising from an infection of the blood in consequence of local suppuration which differs very materially from that just considered. It proceeds from the absorption of putrid matter after air has been admitted into abscesses, but not the introduction of organised pus. Beraud distinguishes it by the title of *putrid infection* of the blood. It is best exemplified in lumbar abscess. The constitution gradually fails under this course of irritation, but the effects are not so rapidly fatal as in *purulent* infection. The former may indeed be cured by removing the cause, and judicious treatment, but the latter generally bids defiance to all curative measures, nor have we any data for determining what line of therapeutical conduct will diminish the almost certain fatality which attends it.

We have been highly gratified by a perusal of Dr. Hun's memoir, and have endeavored, in the foregoing analysis, to give a synopsis of the principal points of interest and practical value.

